# Evaluation of Extra-High Translucent Dental Zirconia: Translucency, Crystalline Phase, Mechanical Properties, and Microstructures †

**DOI:** 10.3390/jfb16010013

**Published:** 2025-01-03

**Authors:** Hiroto Nakai, Masanao Inokoshi, Hengyi Liu, Motohiro Uo, Manabu Kanazawa

**Affiliations:** 1 Department of Gerodontology and Oral Rehabilitation, Graduate School of Medical and Dental Sciences, Institute of Science Tokyo, Tokyo 113-8549, Japan; 2 Department of Oral Devices and Materials, Graduate School of Medical and Dental Sciences, Institute of Science Tokyo, Tokyo 113-8549, Japan; 3 Oral Science Center, Institute of Science Tokyo, Tokyo 113-8549, Japan; 4 Hospital of Stomatology, Guanghua School of Stomatology, Guangdong Provincial Key Laboratory of Stomatology, Sun Yat-sen University, Guangzhou 510055, China; 5 Department of Advanced Biomaterials, Graduate School of Medical and Dental Sciences, Institute of Science Tokyo, Tokyo 113-8549, Japan; 6 Clinic of General, Special Care and Geriatric Dentistry, Center for Dental Medicine, University of Zurich, Plattenstrasse 11, 8032 Zurich, Switzerland

**Keywords:** zirconia, yttria-stabilized zirconia (YSZ), X-ray diffraction (XRD), Rietveld refinement, biaxial flexural strength, crystalline phase, Weibull analysis, microstructural analysis, average grain size, grain size distribution

## Abstract

Highly translucent zirconia (TZ) is frequently used in dentistry. The properties of several highly translucent zirconia materials available in the market require an in-depth understanding. In this study, we assessed the translucency, crystalline phase, mechanical properties, and microstructures of three newly developed highly translucent zirconia materials (Zpex 4. m, 4 mol% yttria-stabilized zirconia: 4YSZ; Zpex Smile.m, 5YSZ; ZR Lucent ULTRA, 6YSZ). The translucency parameter (TP) was analyzed using the CIELAB system. X-ray diffraction was conducted for the crystalline phase analysis, followed by Rietveld refinement. A biaxial flexural strength test using the Weibull analysis was performed to evaluate the mechanical properties. Scanning electron microscopy, grain size distribution, and average grain size were used to analyze the microstructures. The TP content of the ZR Lucent ULTRA was the highest among the samples investigated. The Rietveld analysis revealed that the cubic zirconia phase content of the ZR Lucent ULTRA was the highest. The biaxial flexural strength of the ZR Lucent ULTRA was the lowest (622.9 MPa). The average grain size and proportion of large grains (1.0 µm < x) were the highest in ZR Lucent ULTRA. Therefore, extra-high translucent zirconia has the potential for use in anterior monolithic restorations owing to its esthetics and strength.

## 1. Introduction

Zirconia ceramics offer excellent mechanical properties and biocompatibility, and they are superior to metallic materials in terms of esthetic uses [1,2,3]. In contrast to metals, zirconia ceramics offer the advantage of being non-allergenic. Currently, zirconia ceramics are widely used in dental prostheses, such as crowns, bridges, inlays, veneers, implant bodies, and implant abutments [3,4,5]. Initially, zirconia ceramics were considered to cause significant tooth structure wear because of their hard polycrystalline nature. However, compared to other ceramic materials, monolithic zirconia causes minimal wear of antagonists, especially if appropriately polished [6,7,8]. Restorations fabricated from monolithic zirconia materials show relatively low fracture rates in short-term evaluations [9,10].

Zirconia ceramics used in dental products consist of polycrystals with monoclinic, tetragonal, and cubic phases. Zirconia is the most stable monoclinic phase at room temperature [11]. The mechanical strength of the monoclinic phase was lower than that of the tetragonal phase. The properties of the tetragonal zirconia are closely related to zirconia. The stress-activated tetragonal-to-monoclinic zirconia phase transformation inhibits crack growth, which toughens the zirconia ceramics [12,13]. The addition of yttria to zirconia polycrystals stabilizes the tetragonal phase even at room temperature [11]. Conventionally, 3 mol% yttria-stabilized tetragonal zirconia polycrystals (3Y-TZP) have been developed and are typically used in dental zirconia products [3]. Regarding optical properties, conventional 3Y-TZP containing 0.25 wt% Al_2_O_3_ shows high opacity. The translucency of the highly translucent 3Y-TZP was determined to a certain degree by varying the Al_2_O_3_ content (0.1 or 0.05 wt%). However, highly translucent 3Y-TZP cannot be used in anterior fixed dental prostheses (FDPs) because of its low translucency [14,15].

Highly translucent yttria-stabilized zirconia (YSZ) with a high yttria content has received significant attention owing to its superior optical properties compared to 3Y-TZP. Highly translucent YSZ improves translucency by adding 4 mol% or more yttria to zirconia polycrystals, as higher yttria content results in higher cubic-phase zirconia content [16]. Porcelain-fused-to-metal (PFM) and porcelain-fused-to-zirconia (PFZ) FDPs are widely used primary options for anterior restorations. However, these materials exhibit chipping problems when used as veneering ceramics [17,18,19]. Monolithic zirconia restorations can prevent the chipping of veneering ceramics and have the advantage of reducing the amount of preparation for abutment teeth compared with PFM and PFZ restorations. To apply highly translucent zirconia in anterior monolithic restorations, it is essential to verify whether the YSZ has sufficient optical and mechanical properties.

Therefore, this study aims to investigate the influence of different yttria contents on the translucency, crystalline phase, mechanical properties, and microstructures of YSZ. The null hypothesis was that the yttria content of YSZ does not affect the translucency, crystalline phase, mechanical properties, or microstructures of highly translucent zirconia. This study is an expanded version of a previous study [20].

## 2. Materials and Methods

Three YSZ grades were investigated: (1) Zpex 4.m (4YSZ, Tosoh, Tokyo, Japan), (2) Zpex Smile.m (5YSZ, Tosoh, Tokyo, Japan), and ZR Lucent ULTRA (6YSZ, Shofu, Kyoto, Japan). All the specimens were prepared in a disk shape with a diameter of 14.5 mm and a thickness of 1.2 mm after sintering (total number of specimens = 23 per group). Mirror-like polished specimens were used for the translucency measurements and biaxial flexural strength measurements (*n* = 20 per group). The specimens for crystalline phase and microstructural analysis were sintered without grinding or polishing (*n* = 3 per group). The sintering protocols used are listed in Table 1.

The manufacturing procedure for Zpex 4. m, and Zpex smile.m specimens was as follows: the powders consisted of Zpex 4. m and Zpex smile and were pressed at 390 kN for 60 s and cold isostatically pressed at 200 MPa for 60 s to obtain the zirconia blocks. After degreasing at 500 °C for 44 h and hand temporary sintering at 985 °C for 36 h, green bodies were subtractively manufactured in a cylindrical shape using a milling machine (DGShape DWX-52DCi, Roland DGA Corp, Irvine, CA, USA). Disk-shaped specimens were cut using a cutting machine (Secotom; Struers, Ballerup, Denmark). After sintering, they were ground using a surface grinding machine (GRIND-X PFG500II, Okatomo, Gunma, Japan), and mirror-like polished using polishing paste (Zir-Gross, Shofu, Kyoto, Japan) and a polishing brush (Pivot-Brush HP, Shofu).

The manufacturing procedure of the ZR Lucent ULTRA was as follows: the zirconia block of Disk ZR Lucent ULTRA was subtractively manufactured into disk-shaped specimens using a milling machine (DGShape DWX-52DCi, Roland DGA Corp.). Disk-shaped specimens were cut using a cutting machine (Secotom; Struers, Ballerup, Denmark). After sintering, the grinding and polishing procedures were identical to those described above.

### 2.1. Translucency

A colorimeter (CR-20; Konica Minolta Sensing, Tokyo, Japan) was used to analyze the translucency parameter (TP) based on the CIELAB system. The specimens (*n* = 7 per group) were measured on white (CIE L* = 95.4, a* = −1.3, b* = −2.4) and black (CIE L* = 18.9, a* = −0.5, b* = −2.5) plates with the coupling medium (glycerin) placed between plates and specimens [21]. The TP was calculated using the following formula: TP = [(L_B_ − L_W_)^2^ + (a_B_ − a_W_)^2^ + (b_B_ − b_W_)^2^]^1/2^, where the subscripts W and B refer to the color coordinates of the white and black plates, respectively.

### 2.2. Crystalline Phase Analysis

To clarify the zirconia phase composition of YSZ, X-ray diffraction (XRD; Rigaku Miniflex 600, Rigaku, Tokyo, Japan) was conducted with Cu Kα (40 kV, 15 mA) at 0.02°/2 s in the range of 20–90° (*n* = 3 per group). The relative phase contents of cubic zirconia (*c*-ZrO_2_), tetragonal zirconia (*t*-ZrO_2_), tetragonal prime zirconia (*t’*-ZrO_2_), and monoclinic zirconia (*m*-ZrO_2_) were determined via Rietveld analysis using TOPAS Academic V7 software (Coelho software, Brisbane, Australia). The Y_2_O_3_ content of *t*-ZrO_2_ was calculated using the equation proposed by Krogstad et al. [22].

### 2.3. Biaxial Flexural Strength

A biaxial flexural strength test was conducted to assess the flexural strength using a universal testing machine (E.Z. test, Shimadzu Corporation, Tokyo, Japan) with the piston-on-three-ball method following 6872 (2015). Specimens (*n* = 20 per group) were loaded at a crosshead speed of 0.5 mm/min until failure. The Poisson’s ratio of the zirconia grades was set to 0.3, as previously described [23].

### 2.4. Microstructure

The surface of each specimen (*n* = 1 per group) was analyzed using scanning electron microscopy (SEM: JSM-7900F, JEOL, Tokyo, Japan) at an accelerating voltage of 5 kV, an emission current of 8 µA, and a working distance of 10 mm. Prior to the observation, the specimens were coated with a thin layer of Pt (E102 ion sputtering; Hitachi, Tokyo, Japan). To calculate the grain size distribution, more than 1800 grains were investigated for each specimen group. The grains were classified by their size following the methods employed by Liu et al. with the following modifications: fine (x ≤ 0.2 μm); medium (0.2 µm < x ≤ 1.0 μm); and large (1.0 µm < x). The linear intercept method was used to analyze the average grain size. ImageJ software (version 1.54g, National Institutes of Health, Bethesda, MD, USA) was used to investigate the grain size distribution and average grain size.

### 2.5. Statistical Analysis

For the statistical analysis of translucency and average grain size, the Shapiro–Wilk test was conducted to assess data distribution followed by either a one-way analysis of variance (ANOVA) with a Tukey’s post hoc test or the Kruskal–Wallis test with a Dunn’s test. Flexural strength data were statistically analyzed using the Weibull analysis. The Weibull parameters were calculated using the maximum-likelihood estimation. The likelihood ratio was used to calculate the confidence interval bounds. Additionally, the likelihood contour method was employed to determine the statistical differences between Weibull distributions [24]. Using the likelihood contour method, the intersecting contour plots indicated no significant differences. In addition, Pearson’s correlation coefficients were estimated to evaluate the relationship between the five factors: TP, tetragonal phase content, cubic phase content, biaxial flexural strength, average grain size, tetragonality, and Y_2_O_3_ (mol%) in the tetragonal phase. All analyses were conducted at a significant level of α = 0.05 using software packages R4.4.2 and weibullR (R Foundation for Statistical Computing, Vienna, Austria).

## 3. Results

### 3.1. Translucency

**Figure 1:**
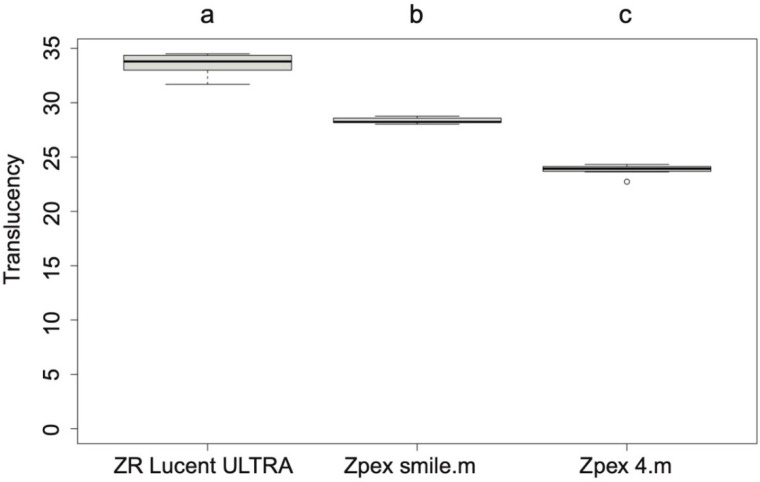
Translucency parameters (TPs) of the highly translucent YSZ products. Different letters indicate a significant difference (*p* < 0.05).

The data were normally distributed based on the Shapiro–Wilk test. One-way ANOVA followed by a Tukey post hoc test revealed that the TP of ZR Lucent ULTRA was statistically the highest (33.5 ± 1.1). The TP of Zpex Smile.m (28.4 ± 0.3) was higher than that of Zpex 4. m (23.8 ± 0.5). Thus, higher yttria content resulted in a higher translucency. These results are shown in Figure 1.

### 3.2. Crystalline Phase Analysis

**Figure 2:**
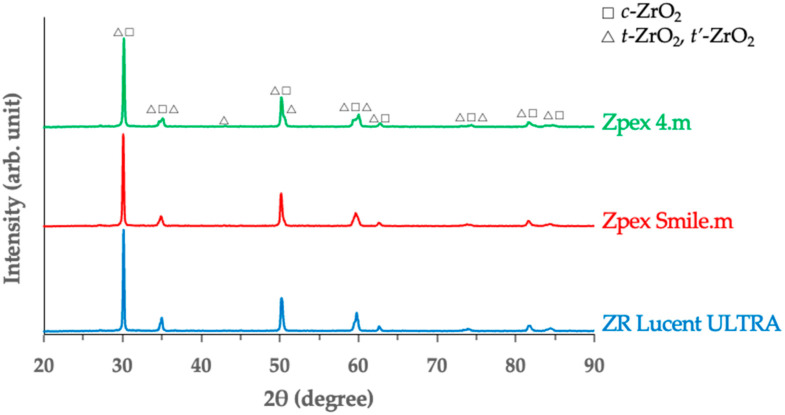
Representative X-ray diffraction (XRD) patterns for the YSZ products.

Representative XRD patterns are shown in Figure 2. The three highly translucent YSZ samples exhibited two peaks corresponding to the tetragonal phase (*t*-ZrO_2_ and *t’*-ZrO_2_). The relative amounts of each phase in YSZ, as determined by a Rietveld analysis, are summarized in Table 2. The ZR Lucent ULTRA contained a higher proportion of *c*-ZrO_2_ than the Zpex Smile.m. By contrast, Zpex 4.m contained a lower proportion of *c*-ZrO_2_ than Zpex Smile.m. The higher the yttria content in YSZ, the greater the proportion of *t’*-ZrO_2_ and *c*-ZrO_2_ phases.

### 3.3. Biaxial Flexural Strength

**Figure 3:**
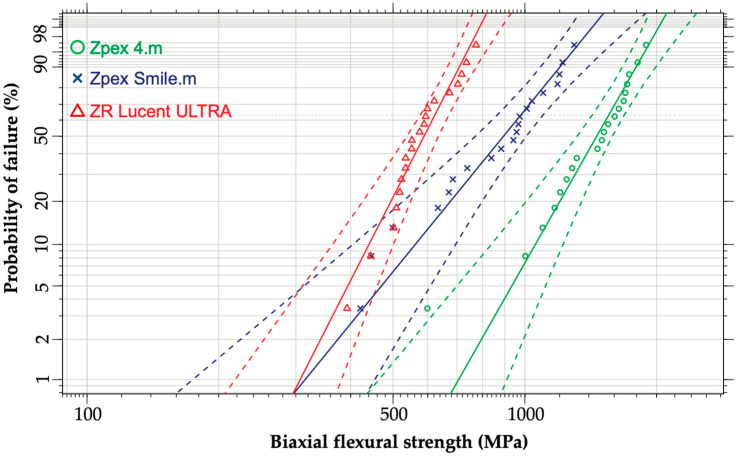
Weibull plots for the YSZ products.

**Figure 4:**
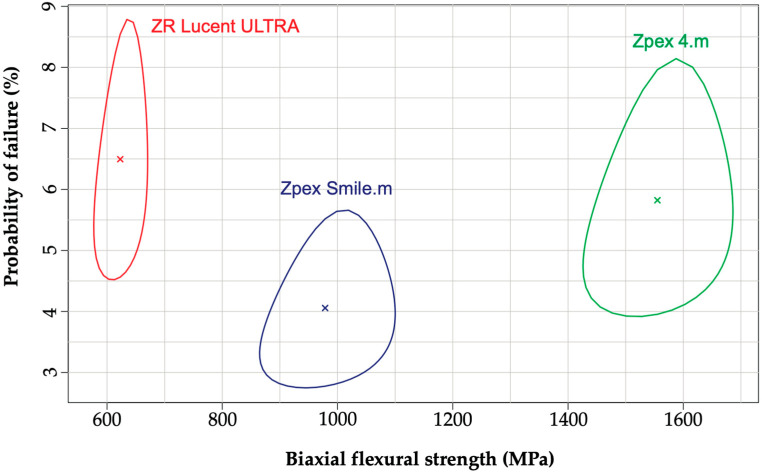
Weibull contour plots (95% confidence intervals) for the YSZ products. The Weibull modulus and characteristic strength of each ceramic grade are presented with “×” inside of the contour plots.

The Weibull analysis results are listed in Table 3. The Weibull distribution and its contour plots for the biaxial flexural strength are shown in Figure 3 and Figure 4, respectively. Weibull analysis revealed that Zpex 4. m exhibited a significantly higher flexural strength than Zpex Smile.m. The ZR Lucent ULTRA had a significantly lower characteristic strength among the three highly translucent zirconia samples investigated.

### 3.4. Microstructure

**Figure 5:**
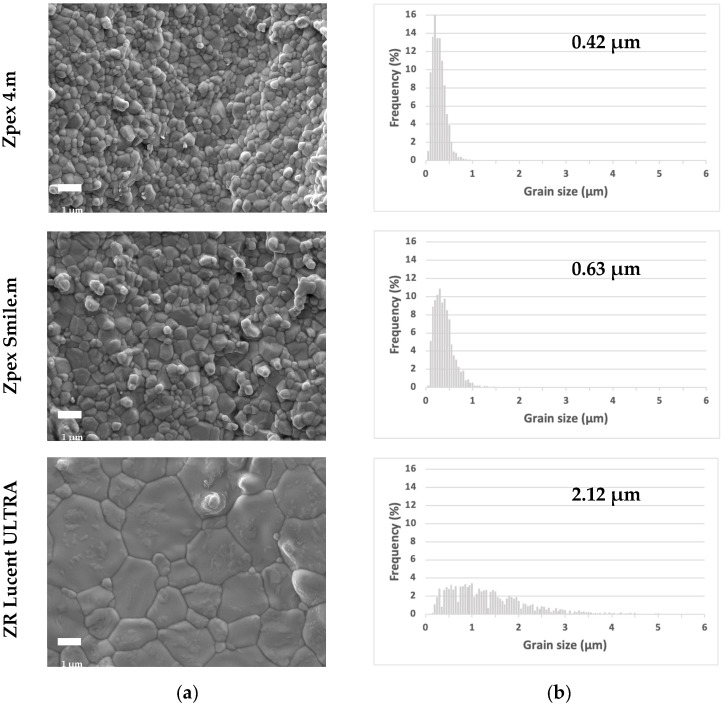
Representative SEM images (**a**) and grain size distribution (**b**).

The grain-size distributions and average grain sizes of the three YSZ samples are summarized in Table 4 and Figure 5. The proportion of fine grains was highest in Zpex 4.m. Conversely, the proportion of large grains was highest in the ZR Lucent ULTRA. The average grain size of the ZR Lucent ULTRA was significantly higher than that of the Zpex Smile.m. Zpex 4.m resulted in a statistically smaller average grain size than that of Zpex Smile. A greater amount of yttria increased the proportion of large grains and the average grain size.

### 3.5. Correlation Analysis

**Figure 6:**
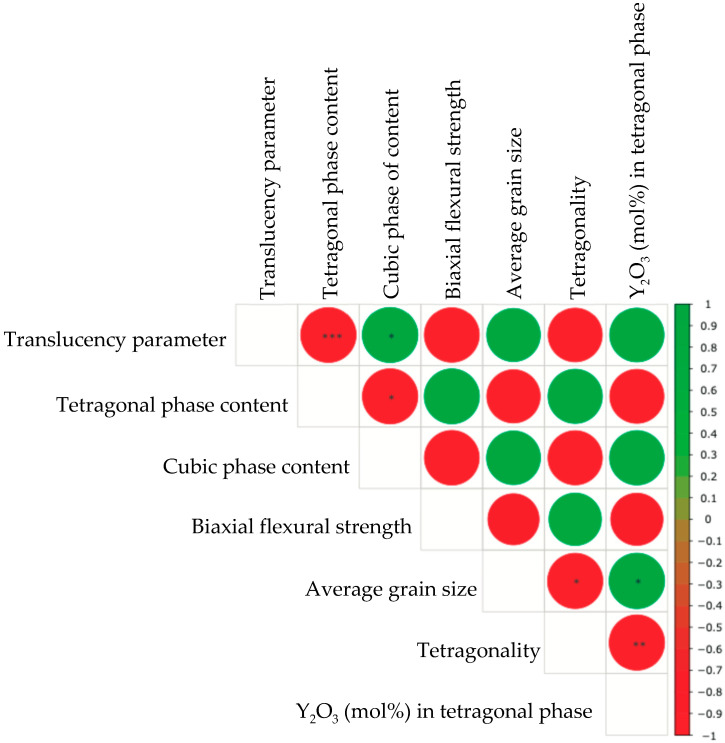
Results of the Pearson’s correlation analysis of each factor. Green and red circles stand for positive and negative correlations, respectively. * *p* < 0.05; ** *p* < 0.01; *** *p* < 0.001.

A summary of the correlation analysis is presented in Figure 6. The TP was positively correlated with the cubic phase content, average grain size, and Y_2_O_3_ (mol%) in the tetragonal phase. The tetragonal phase content was positively correlated with the biaxial flexural strength and tetragonality. The cubic phase content was positively correlated with the average grain size and Y_2_O_3_ content (mol%) in the tetragonal phase. The biaxial flexural strength was positively correlated with tetragonality. The average grain size is positively correlated with Y_2_O_3_ (mol%) in the tetragonal phase. However, the TP was negatively correlated with the tetragonal phase content, biaxial flexural strength, and tetragonality. The tetragonal phase content was negatively correlated with the cubic phase content, average grain size, and Y_2_O_3_ content (mol%) in the tetragonal phase. The cubic phase content was negatively correlated with the biaxial flexural strength and tetragonality. The biaxial flexural strength was negatively correlated with the average grain size and Y_2_O_3_ content (mol%) in the tetragonal phase. The average grain size was negatively correlated with tetragonality. The tetragonality was negatively correlated with Y_2_O_3_ (mol%) in the tetragonal phase.

## 4. Discussion

This study investigated the influence of different yttria contents on the translucency, crystalline phase, mechanical properties, and microstructures of highly translucent YSZ. The TP of the ZR Lucent ULTRA was significantly higher than that of the Zpex Smile.m, which was significantly higher than that of the Zpex 4. m. XRD with Rietveld analysis revealed that ZR Lucent ULTRA contained a higher proportion of *c*-ZrO_2_ than Zpex Smile.m, which in turn contained a higher proportion of *c*-ZrO_2_ than Zpex 4. m. A Weibull analysis revealed that the Zpex 4. m showed a significantly higher flexural strength than Zpex Smile.m, which had a higher flexural strength than ZR Lucent ULTRA. A microstructural analysis revealed that the Zpex 4.m exhibits the highest proportion of fine grains, whereas the ZR Lucent ULTRA exhibits the highest proportion of large grains. Based on these results, the null hypothesis that the yttria content of YSZ does not affect the translucency, crystalline phase, mechanical properties, or microstructures of highly translucent zirconia was rejected.

The TP was used to assess the optical properties in the current study. The TP is defined as the color difference between the reflected colors of a material with a stated thickness backed by black and white backgrounds [21]. The TP is related to human visual perception and is therefore suitable for assessing the translucency of dental restorations. In the present study, the ZR Lucent ULTRA exhibited the highest translucency. Zirconia, which has a high yttria content, exhibits a high translucency owing to its cubic phase. Inokoshi et al. reported that the translucency of KATANA Zirconia UTML (6YSZ) was the highest (36.7 ± 1.8) and that of KATANA Zirconia STML (5YSZ) was the second highest (34.2 ± 0.7), whereas that of KATANA Zirconia HT (4YSZ) was the lowest (29.5 ± 0.9) [25]. Their translucency results indicated a similar tendency to ours. Their TP was slightly higher than ours because the thickness of the specimens was 0.5 mm, whereas that of our specimens was 1.2 mm. Liu et al. reported the translucency of Zpex Smile [26]. Our Zpex Smile. m showed a higher TP than Zpex smile. One reason for this difference was the Al_2_O_3_ content. The Al_2_O_3_ content of Zpex Smile.m and Zpex Smile were below 0.02 and 0.05 wt% [27]. A lower Al_2_O_3_ content results in a higher translucency because Al_2_O_3_ induces light scattering, which decreases the translucency [28,29]. The grain size of zirconia is a crucial factor that affects its translucency. In grain diameters of 0.1–1 μm, grain boundary scattering can be observed, whereas, in grain diameters of 1–10 μm, diffuse transmission is observed and the material would be largely translucent [30]. This finding is consistent with the results of this study.

A Rietveld analysis was performed to calculate the phase compositions of *c*-ZrO_2_, *t*-ZrO_2_, and *m*-ZrO_2_. The *c*-ZrO_2_ of the ZR Lucent ULTRA (6YSZ) was larger than that of the Zpex Smile.m (5YSZ), which was larger than that of the Zpex 4. m (4YSZ). Arcila et al. reported that the *c*-ZrO_2_ content of 5YSZ was higher than that of 4YSZ [31]. Li et al. investigated different yttria contents (3.2, 3.6, 4.0, 4.5, 5.0) and reported that 5.0Y resulted in the highest cubic zirconia phase content [12]. Based on these results, the proportion of cubic-phase zirconia is correlated with the yttria content.

The Weibull analysis in the current study revealed that the biaxial flexural strength increased proportionally as the yttria content decreased. Katada et al. measured the biaxial flexural strengths of LAVA Plus (3YSZ), KATANA Zirconia HTML (4YSZ), KATANA Zirconia STML (5YSZ), and LAVA Esthetic (5YSZ) [32]. Their results showed the same tendencies as those observed in our study. Three highly translucent zirconia investigated in the current study (Zpex 4. m, Zpex Smile.m, and ZR Lucent ULTRA) appeared to have sufficient flexural strength for FDPs. For clinical adaptation, flexural strength and the effects of low-temperature degradation (LTD) must be considered. Although Zpex Smile.m showed high translucency in the current study, it had lower Al_2_O_3_ content than Zpex Smile. The reduced Al_2_O_3_ content significantly lowered hydrothermal stability.

Microstructural analysis using the linear intercept method revealed that the average grain sizes of the ZR Lucent ULTRA, Zpex Smile.m, and Zpex 4. m were 2.12 ± 0.27, 0.63 ± 0.07, and 0.42 ± 0.04, respectively. Using the same method, Liu et al. described the average grain sizes of KATANA Zirconia UTML (6YSZ), KATANA Zirconia STML (5YSZ), KATANA HT (4YSZ), Zpex Smile (5YSZ), and Zpex 4 (4YSZ) as being 2.10 ± 0.38, 1.47 ± 0.23, 0.56 ± 0.06, 0.91 ± 0.09, and 0.67 ± 0.07 [26]. A higher yttria content increased the average grain size in the current study as well as in their results. Regarding the grain size distribution of 5YSZ, KATANA Zirconia STML had a higher proportion of large grains than Zpex Smile and Zpex Smile.m, potentially owing to differences in the sintering temperature. KATANA Zirconia STML was sintered at 1550 °C, whereas Zpex Smile and Zpex Smile.m were sintered at 1450 °C and 1450–1500 °C, respectively. Inokoshi et al. reported that higher sintering temperatures increase the ZrO_2_ grain size [33].

A correlation analysis revealed that the TP was positively correlated with the cubic-phase content. The cubic crystalline phase is isotropic, indicating that its properties are the same in all crystallographic directions, leading to high translucency [34]. The cubic phase content was negatively correlated with the biaxial flexural strength. Zhang et al. reported that a higher cubic content resulted in a lower fracture toughness [27]. The TP was positively correlated with the average grain size. This is because the larger average grain size contained a higher amount of *c*-ZrO_2_.

## 5. Conclusions

A higher yttria content increased the TP, cubic zirconia phase, and average grain size but decreased the flexural strength. The biaxial flexural strengths of Zpex Smile.m (5YSZ) and ZR Lucent ULTRA (6YSZ) were 978.6 and 622.9 MPa, respectively. The results of the current study indicate that extra-high translucent zirconia has the potential for monolithic anterior restorations.

## Figures and Tables

**Table 1:** Sintering protocols for the YSZ products.

Zirconia Grades	Heating Rate	Sintering	Cooling Rate
Zpex 4.m	10 °C/min (1450–1500 °C)	1450–1500 °C for 120 min	natural cooling
Zpex Smile.m	10 °C/min (1450–1500 °C)	1450–1500 °C for 120 min	natural cooling
ZR Lucent ULTRA	60 °C/min, (1000 °C) 3 °C/min, (1550 °C)	1550 °C for 60 min	natural cooling

**Table 2:** Relative amounts of each phase for the YSZ investigated.

Zirconia Grades	Phase Composition (wt%)				Tetragonality	Y_2_O_3_ (mol%) in *t*-ZrO_2_	Goodness of Fit
	*t*-ZrO_2_	*t’*-ZrO_2_	*c*-ZrO_2_	*m*-ZrO_2_			
Zpex 4.m	55.3	40.2	3.5	1.1	1.0151	2.75	1.0
Zpex Smile.m	27.7	58.3	12.7	1.3	1.0148	2.85	1.0
ZR Lucent ULTRA	6.3	68.9	24.2	0.6	1.0136	3.28	1.1

**Table 3:** Summary of the Weibull biaxial strength analysis.

Zirconia Grades	Shape (Modulus)	95% Confidence Level at Modulus	Scale (B63.2)	95% Modulus Level at B63.2 *
Zpex 4.m	5.8	3.7–8.0	1555.1	1425.9–1686.2 ^a^
Zpex Smile.m	4.1	2.6–5.5	978.6	864.9–1100.1 ^b^
ZR Lucent ULTRA	6.5	4.3–8.6	622.9	577.0–670.4 ^c^

* Different letters indicate significant differences (*p* < 0.05).

**Table 4:** Summary of average grain size and grain size distribution of the YSZ products.

Zirconia Grades	Average Grain Size (µm) *	Grain Size Distribution (%)		
		Fine Grains	Medium Grains	Large Grains
Zpex 4.m	0.42 ± 0.04 ^a^	40.27	59.73	0
Zpex Smile.m	0.63 ± 0.07 ^b^	23.75	75.5	0.74
ZR Lucent ULTRA	2.12 ± 0.27 ^c^	1.15	43.11	55.74

* Different letters indicate significant differences (*p* < 0.05).

## Data Availability

The original contributions presented in the study are included in the article; further inquiries can be directed to the corresponding author.
